# Altered expression of differential gene and lncRNA in the lower thoracic spinal cord on different time courses of experimental obstructive jaundice model accompanied with altered peripheral nociception in rats

**DOI:** 10.18632/oncotarget.22532

**Published:** 2017-11-20

**Authors:** Qian Wang, Zhi-Xiao Li, Bao-Wen Liu, Zhi-Gang He, Cheng Liu, Min Chen, San-Guang Liu, Wei-Zhong Wu, Hong-Bing Xiang

**Affiliations:** ^1^ Department of Anesthesiology and Pain Medicine, Tongji Hospital, Tongji Medical College, Huazhong University of Science and Technology, Wuhan, P.R. China; ^2^ Department of Anesthesiology, Hubei Maternal and Child Health Hospital, Wuhan, P.R. China; ^3^ Department of Hepatobiliary Surgery, The Second Hospital, Hebei Medical University, Shijiazhuang, P.R. China; ^4^ Department of General Surgery, The Second Hospital, Hebei Medical University, Shijiazhuang, P.R. China

**Keywords:** obstructive jaundice, nociceptive response, high-throughput sequencing, lncRNA, spinal cord

## Abstract

The spinal origin of jaundice-induced altered peripheral nociceptive response poorly understood. In the current study, we aimed to first validate rats with bile duct ligation (BDL) as a jaundice model accompanied by altered peripheral nociceptive response, and then to analyze differential gene and lncRNA expression patterns in the lower thoracic spinal cord on different time courses after BDL operation by using high-throughput RNA sequencing. The differentially expressed genes (DEGs) identified using reverse transcription-quantitative polymerase chain reaction (RT-qPCR) analysis, followed by clustering analysis, Gene Ontology analysis and pathway analysis. As a result, a total of 2033 lncRNAs were differentially expressed 28d after BDL, in which 1545 probe sets were up-regulated and 488 probe sets were down-regulated, whereas a total of 2800 mRNAs were differentially expressed, in which 1548 probe sets were up-regulated and 1252 probe sets were down-regulated. The RNAseq data of select mRNAs and lncRNAs was validated by RT-qPCR. 28d after BDL, the expressions of lncRNA NONRATT002335 and NONRATT018085 were significantly up-regulated whereas the expression of lncRNA NONRATT025415, NONRATT025388 and NONRATT025409 was significantly down-regulated. 14d after BDL, the expressions of lncRNA NONRATT002335 and NONRATT018085 were significantly up-regulated; the expression of lncRNA NONRATT025415, NONRATT025388 and NONRATT025409 was significantly down-regulated. In conclusion, the present study showed that jaundice accompanied with decreased peripheral nociception involved in the changes of gene and lncRNA expression profiles in spinal cord. These findings extend current understanding of spinal mechanism for obstructive jaundice accompanied by decreased peripheral nociception.

## INTRODUCTION

Obstructive jaundice is a clinical syndrome related to bile cholestasis and is governed by complex signals [1–3]. While historically studied by researching the obstructive jaundice, inputs from the “jaundiced live” and cholestasis impact many functional conditions [4–6]. Indeed, emerging data suggest that significant progress has been made in understanding the communication between the cholestatic liver diseases and spinal cord in jaundice regulation [7–10]. The spinal cord is well known to be a complex community of specific genes and long noncoding RNAs (lncRNAs) [11–13] that profoundly influence many aspects of occurrence and development for pathologic disorders [14–20], including development of the jaundice regulation. Though there are various studies in clinical disorders by obstructive jaundice, its differential gene and lncRNA expression pattern in the thoracic spinal cord remains unknown.

The introduction of high-throughput RNA sequencing (RNA-seq), where thousands of differential genes and lncRNA expression patterns can be studied in parallel [21–24], permits a broad assessment for altered expression of differential gene and lncRNA in the lower thoracic spinal cord on different time courses of obstructive jaundice model. In current study, we performed a comprehensive transcriptome analysis in obstructive jaundice model using RNA-seq, and identified lncRNAs with differential expression. To explore the function of lncRNAs, we predicted their potential targets with cis-regulatory effects, which were then put into gene ontology (GO) and Kyoto Encyclopedia of Genes and Genomes (KEGG) for further analysis. In addition, we compared the expression of some lncRNAs in thoracic spinal cord at different time points in obstructive jaundice model.

## RESULTS

### Evaluation of jaundice

**Figure 1:**
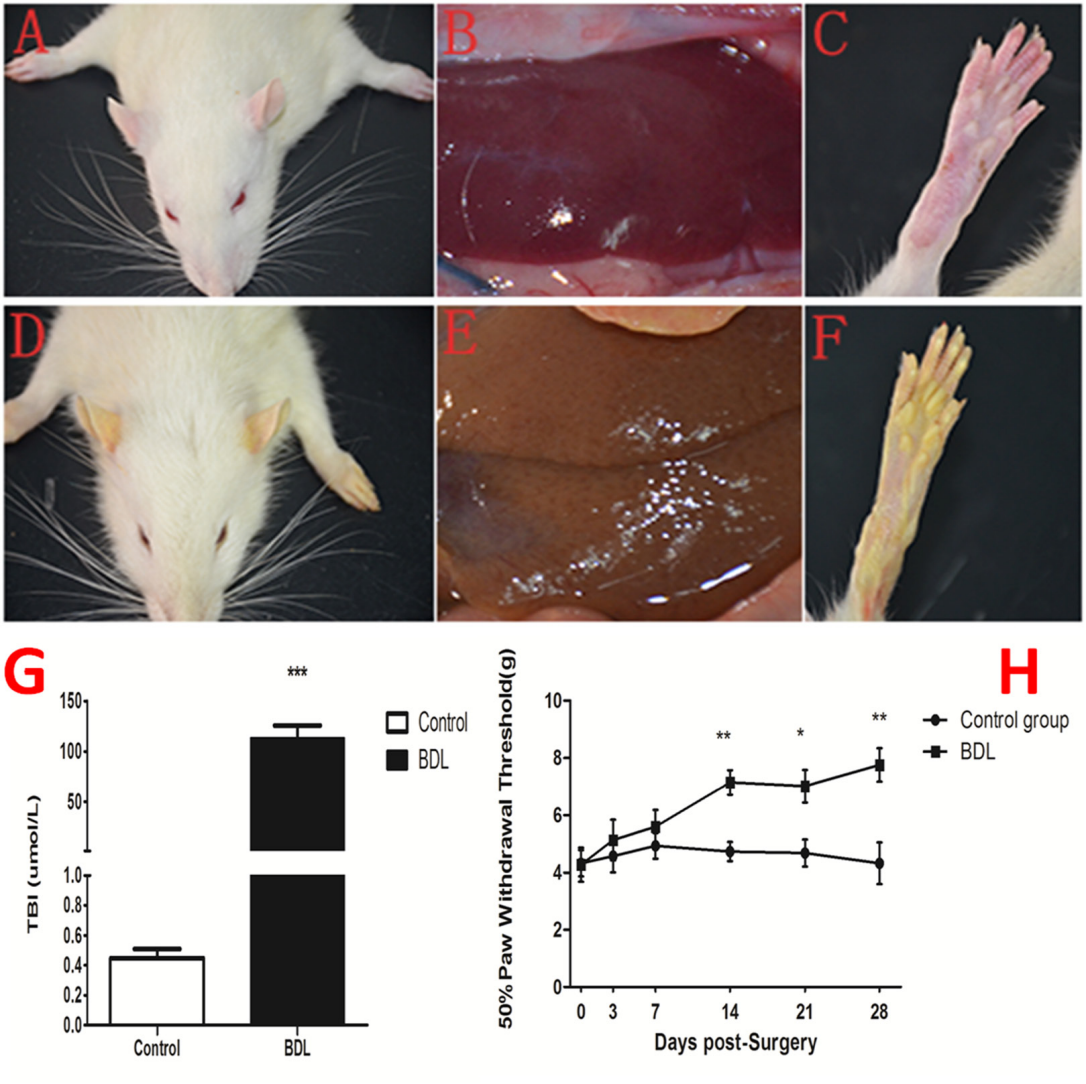
Evaluation of jaundice and the change of nociceptive threshold following BDL operation **(A-F)** The rats in BDL group showed yellow ears (D), liver tissue(E) and rear claw(F) 28 days after surgery compared to control group (A, B, C). **(G)** The serum total bilirubin levels was significantly higher in the BDL group (112.8±12.88μml/L) than in the control group (0.45±0.06μml/L). **(H)** The mechanical PWT was tested to assess the jaundice-induced nociceptive threshold change prior to (day 0) and at days 3, 7, 14, 21, and 28 following BDL operation. The hindpaw of BDL rats displayed a dramatic decrease in mechanical PWT to von Frey filament stimulation from 4.28±0.59 g at baseline to 7.15 ±0.43 g 14d after BDL operation and further deteriorated to 7.02 ± 0.57 at day 21, and 7.76 ±0.58g at day 28. ^*^P<0.05, ^**^P<0.01, ^***^P<0.001 compared with the each corresponding time point.

We observed the general appearance of the animals used in this study (Figure 1), and found that rats in BDL group showed yellow ears (D), liver tissue(E) and rear claw(F) 28 days after surgery compared to control group (A, B, C). Otherwise, our results showed that the serum total bilirubin levels was significantly higher in the BDL group (112.8±12.88μml/L) than in the control group (0.45±0.06μml/L) (Figure 1G).

### The change of nociceptive threshold following BDL operation

The mechanical nociceptive stimulation was tested to assess the jaundice-induced nociceptive threshold change prior to (day 0) and at days 3, 7, 14, 21, and 28 following BDL operation. Our result showed that the hindpaw of BDL rats displayed a dramatic increase in mechanical nociceptive threshold to von Frey filament stimulation from 4.28±0.59 g at baseline to 7.15 ±0.43 g 14d after BDL operation and further deteriorated to 7.02 ± 0.57 at 21d and 7.76 ±0.58g at 28d after BDL operation (Figure 1H). These data indicated that obstructive jaundice resulted in the decreased sensitivity in response to the mechanical nociceptive stimulation in the present study.

### Differentially expressed genes of T6-T12 spinal cord 28d after BDL operation

**Figure 2:**
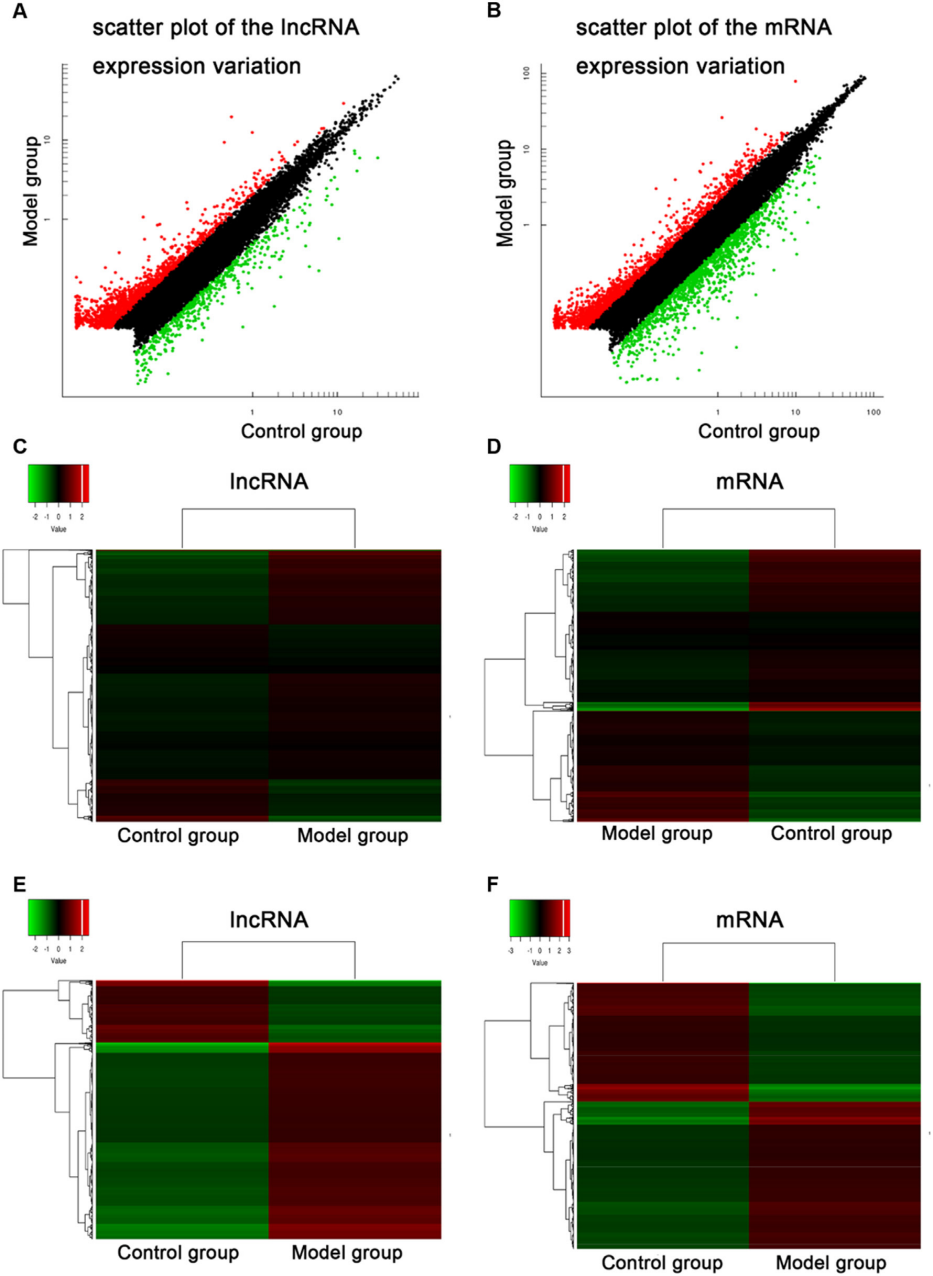
Common bile duct ligation resulted in the expression profiling changes of lncRNA and mRNA Scatter plot comparing global mRNA **(A)** gene expression profiles in the spinal cord between BDL group (Model group) and sham group (Control group) rats; Red color is indicative of up-regulated and green color is indicative of down-regulated genes. Black color is indicative of not statistical significant different genes when it does not pass the cutoff values of 1 and −1 in log2 scale. Heat map showing hierarchical clustering of overall mRNA **(B)** expression pattern of reliably measured probe sets. Heat map showing hierarchical clustering of mRNA **(C)**, whose expression changes were more than twofold. In clustering analysis, up- and down-regulated genes are colored in red and green, respectively.

To systematically identify jaundice-associated lncRNAs in spinal cord, the differential expression analysis was performed between BDL group and control group. The lncRNA and mRNA expressions of T6-T12 spinal cord of the animals were examined the HiSeq 2000 system (Illumina, Inc.) with a total of 1,198,903,526 raw reads from the nine libraries. Differentially expressed gene profiles in BDL group were compared to the corresponding data from control group. Figure 2 indicated the scatter plot comparing global mRNA (A) gene expression profiles in the spinal cord between BDL group (Model group) and sham group (Control group), and heat map showing hierarchical clustering of overall mRNA (B) expression pattern of reliably measured probe sets. In addition, heat map also showed hierarchical clustering of mRNA (C), whose expression changes were more than twofold.

We identified that a total of 2033 lncRNAs were differentially expressed between control group and BDL group, in which 1545 probe sets were up-regulated and 488 probe sets were down-regulated, whereas a total of 2800 mRNAs were differentially expressed between control group and BDL group, in which 1548 probe sets were up-regulated and 1252 probe sets were down-regulated. The detail information of the top 20 up-regulated and 20 down-regulated lncRNAs was in Table 1, and that of the top 20 up-regulated and 20 down-regulated mRNAs was in Table 2. The maximal and minimal fold change was 35.01 and 2.01, respectively.

**Table 1:** The detail information of the top 20 up-regulated and 20 down-regulated lncRNAs

Up-regulatedlncRNAs	Log_2_ fold change(Model/control)	Down-regulatedlncRNAs	Log_2_ fold change(Model/control)
*NONRATT025327*	35.01176765	*NONRATT025415*	−27.18649832
*NONRATT000845*	22.26444654	*NONRATT025388*	−22.79828
*NONRATT002335*	20.87539552	*NONRATT025409*	−18.60605684
*NONRATT018085*	20.23490953	*NONRATT025389*	−18.42176407
*gi|672030539|ref|XR_598272.1|*	17.2076069	*NONRATT006517*	−17.6681701
*NONRATT001654*	14.26208272	*NONRATT012834*	−11.53074647
*NONRATT006896*	13.42852291	*NONRATT028806*	−9.221338198
*gi|672021044|ref|XR_347474.2|*	12.94719615	*gi|672015760|ref|XR_599773.1|*	−9.002940997
*NONRATT022152*	12.644969	*gi|672089057|ref|XR_597824.1|*	−8.581978626
*NONRATT030272*	12.62142301	*NONRATT018084*	−7.331131967
*gi|672019340|ref|XR_601027.1|*	12.55900993	*NONRATT023718*	−6.747783528
*gi|672029209|ref|XR_596791.1|*	11.512809	*NONRATT031185*	−6.642754333
*NONRATT011339*	11.21739711	*NONRATT029420*	−6.473944169
*NONRATT027405*	11.11711302	*NONRATT010390*	−6.470431266
*NONRATT028521*	10.78646213	*NONRATT020020*	−6.22851892
*NONRATT006730*	10.55666403	*NONRATT013131*	−6.121677607
*gi|672016774|ref|XR_600156.1|*	10.39316965	*NONRATT006732*	−5.906932924
*gi|672079892|ref|XR_596396.1|*	10.08651117	*NONRATT019209*	−5.848382084
*gi|672024744|ref|XR_599302.1|*	10.0551937	*NONRATT022428*	−5.771813271
*gi|672033238|ref|XR_349371.2|*	9.84667827	*NONRATT022429*	−5.730278642

**Table 2:** The detail information of the top 20 up-regulated and 20 down-regulated mRNAs

Gene symbol	Description	Log_2_ fold change(Model/control)
**Up-regulated genes**		
*Gemin8*	gem (nuclear organelle) associated protein 8	23.14275154
*Serpina3n*	serine (or cysteine) peptidase inhibitor, clade A, member 3N	13.96190308
*Trim63*	tripartite motif containing 63, E3 ubiquitin protein ligase	10.67545524
*Olr728*	olfactory receptor 728	10.23434831
*Hif3a*	hypoxia inducible factor 3, alpha subunit	9.861685919
*Olr666*	olfactory receptor 666	9.644311752
*Olr1206*	olfactory receptor 1206	9.628587408
*Retnla*	resistin like alpha	9.376262216
*Il6st*	interleukin 6 signal transducer	9.164598351
*Retnlb*	resistin like beta	9.109799277
*Cxcl17*	chemokine (C-X-C motif) ligand 17	8.725497102
*Reg3b*	regenerating islet-derived 3 beta	8.598931015
*Olr383*	olfactory receptor 383	8.574541671
*Olr1192*	olfactory receptor 1192	8.391854793
*Olr1075*	olfactory receptor 1075	8.369125332
*Tas2r114*	taste receptor, type 2, member 114	8.360873477
*Zbtb16*	zinc finger and BTB domain containing 16	8.294338675
*RGD1309489*	similar to Est1p-like protein B	8.266135149
*Olr1063*	olfactory receptor 1063	8.240355839
*Olr215*	olfactory receptor 215	8.230039718
**Down-regulated genes**		
*Shisa3*	shisa family member 3	−71.14729446
*Sostdc1*	sclerostin domain containing 1	−47.37907925
*Sfrp4*	secreted frizzled-related protein 4	−32.38831334
*Smoc2*	SPARC related modular calcium binding 2	−29.71653174
*Smoc2*	SPARC related modular calcium binding 2	−25.62228109
*Mfap4*	microfibrillar-associated protein 4	−25.00123131
*Col1a1*	collagen, type I, alpha 1	−20.63943781
*Gxylt2*	glucoside xylosyltransferase 2	−18.03100637
*Col15a1*	collagen, type XV, alpha 1	−17.56597588
*Igfbp5*	insulin-like growth factor binding protein 5	−15.10090131
*Selp*	selectin P	−14.44927067
*Cdh1*	cadherin 1	−14.28981311
*Col12a1*	collagen, type XII, alpha 1	−14.02389161
*Scin*	scinderin	−14.00885112
*Wisp1*	WNT1 inducible signaling pathway protein 1	−13.78032056
*Col6a3*	collagen, type VI, alpha 3	−13.72869989
*Col3a1*	collagen, type III, alpha 1	−13.16577469
*Loxl1*	lysyl oxidase-like 1	−12.37937746
*Smim5*	small integral membrane protein 5	−11.67644662
*Prdm6*	PR domain containing 6	−11.448275

### Gene ontology annotation for differential expression genes

**Figure 3:**
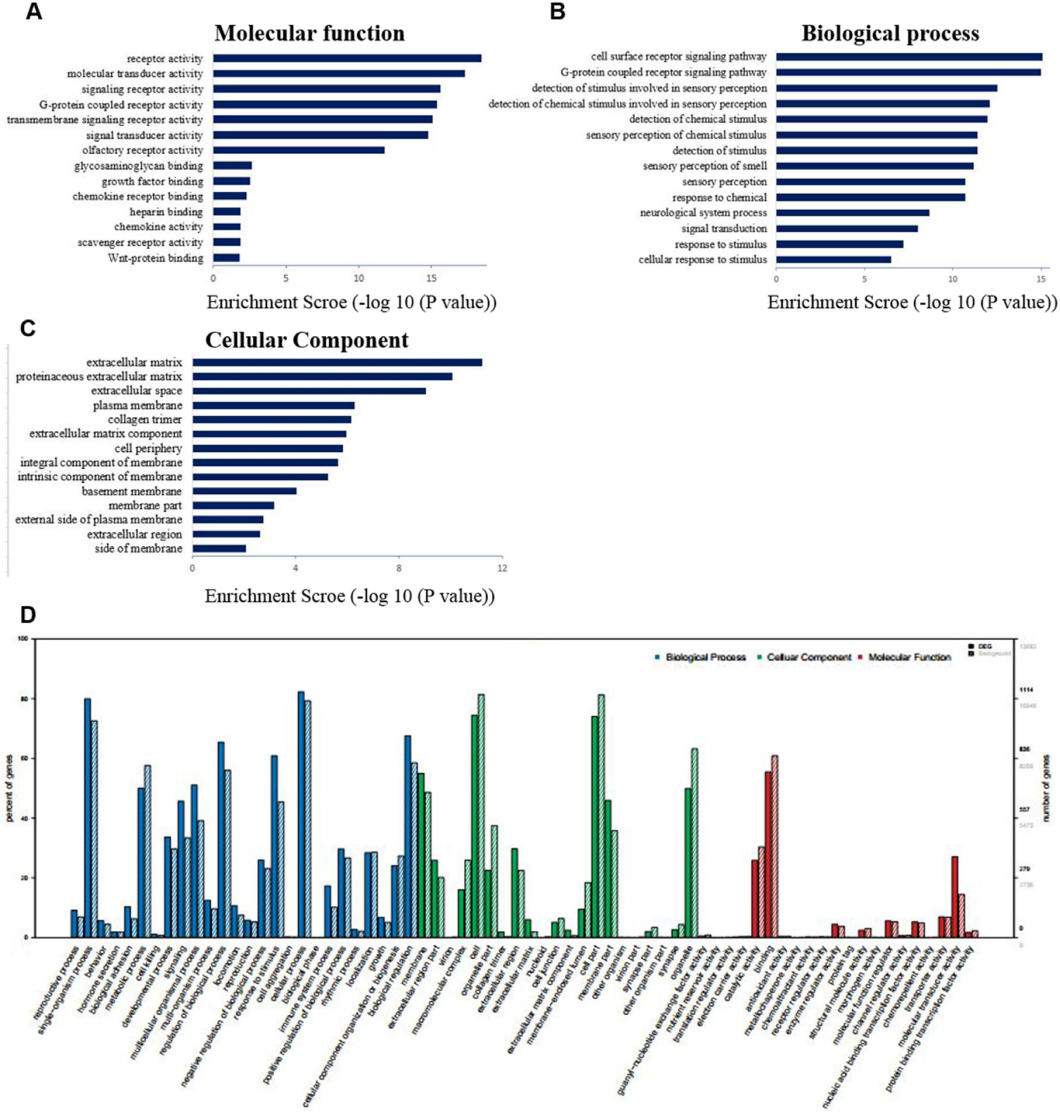
The differential expression of mRNAs in spinal cord after common bile duct ligation-induced jaundice model was analyzed by Gene Ontology (GO) annotation and enrichment **(A)** molecular function classification; **(B)** biological process classification; **(C)** cellular component classification. **(D)** The differential expression genes were analyzed with GO background significant enrichment. Solid bar represents target genes set, slash bar represents genes set.

**Figure 4:**
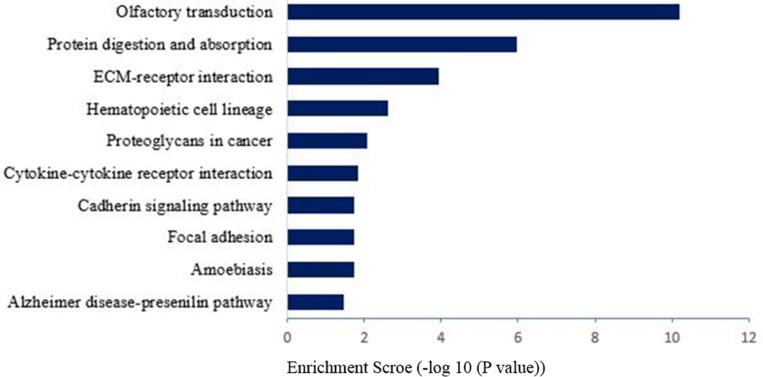
Pathway analysis for differential expression of mRNAs in spinal cord after common bile duct ligation-induced jaundice model

We had used the RNA-seq analyses to identify the differentially expressed genes which were annotated using the GO database (Gene Ontology,http://www.geneontology.org/). The present study showed three important results involved in biological functional groups including molecular function (Figure 3A), biological process (Figure 3B), and cellular component (Figure 3C). Figure 3D indicated that the differential expression genes were analyzed with GO background significant enrichment, and the differentially expressed mRNAs in spinal cord were primarily involved in the biological processes GO functions (Figure 4).

### Validation of the lncRNAs by RT-qPCR 28d after BDL operation

**Figure 5:**
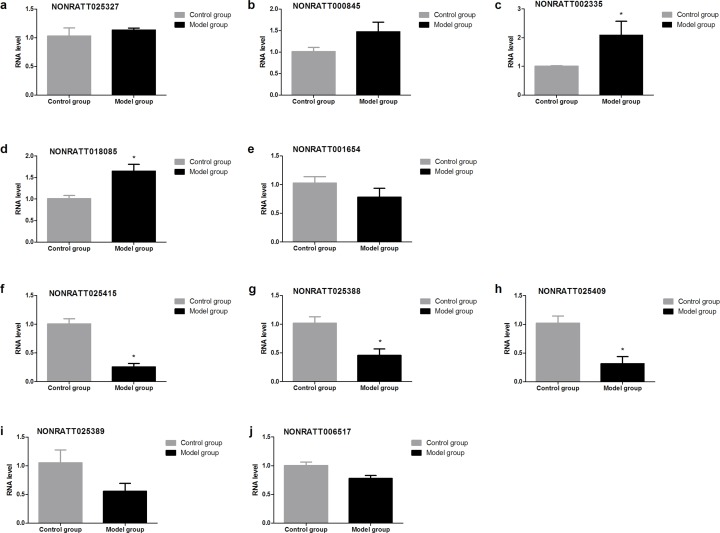
Validation of the lncRNAs by RT-qPCR 28d after BDL operation The expressions of lncRNA NONRATT002335 and NONRATT018085 were significantly up-regulated underlying BDL group compared with control group, whereas the expression of lncRNA NONRATT025415, NONRATT025388 and NONRATT025409 was significantly down-regulated in model group.

To validate the RNA-seq data, top 5 up-regulated lncRNAs (NONRATT025327, NONRATT000845, NONRATT001654, NONRATT002335, and NONRATT018085) and top 5 down-regulated lncRNAs (NONRATT025415, NONRATT025388, NONRATT025409, NONRATT02538 and NONRATT006517) that were differentially expressed between BDL group and control group were randomly selected (Table 3), and their relative expression levels were quantified by RT-qPCR. We found that the expressions of lncRNA NONRATT002335 and NONRATT018085 were significantly up-regulated underlying BDL group compared with control group, whereas the expression of lncRNA NONRATT025415, NONRATT025388 and NONRATT025409 was significantly down-regulated in model group (Figure 5).

**Table 3:** Top 5 up-regulated lncRNAs

ProbeName	log2Fold change (Model group/control group)	GeneID	Product length
NONRATT025327	35.0117676468596	NONRATG020647.2	150
NONRATT000845	22.2644465403801	NONRATG000689.2	212
NONRATT002335	20.8753955158768	NONRATG001912.2	150
NONRATT018085	20.2349095300728	NONRATG014750.2	81
NONRATT001654	14.2620827239878	NONRATG001356.2	132
Top 5 down-regulated lncRNAs			
NONRATT025415	27.1864983217999	NONRATG020725.2	120
NONRATT025388	22.7982799999892	NONRATG020701.2	89
NONRATT025409	18.6060568387294	NONRATG020719.1	111
NONRATT025389	18.4217640674687	NONRATG020702.2	131
NONRATT006517	17.6681700975811	NONRATG005240.2	95

### The expression of 10 lncRNAs in T6-T12 spinal cord at different time points (14d/28d) after BDL operation

**Figure 6:**
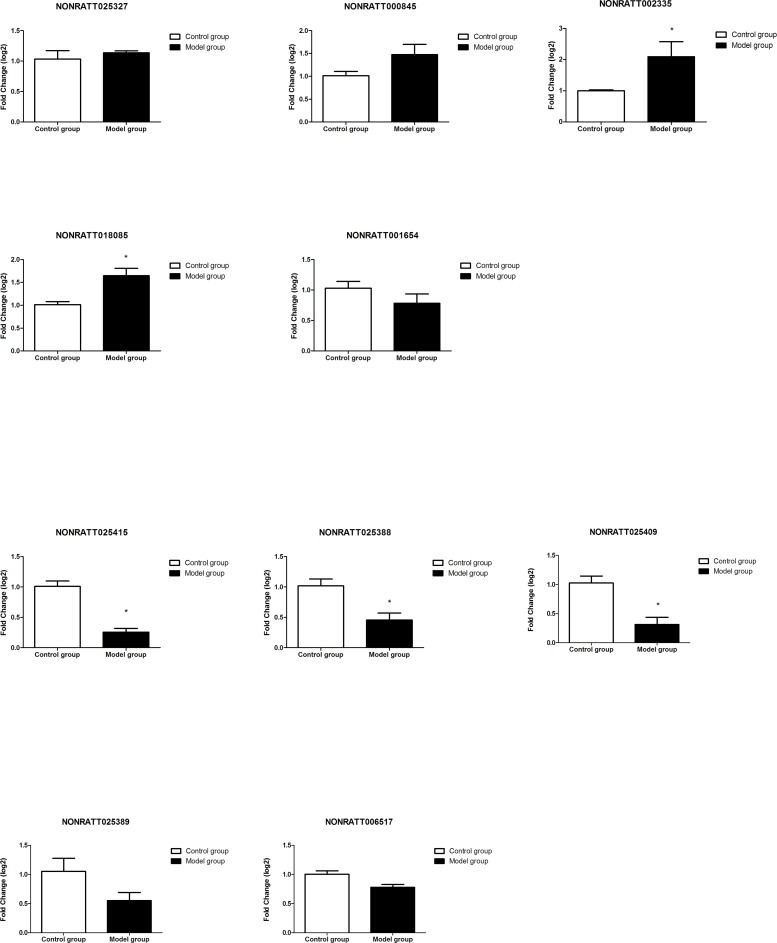
Validation of the lncRNAs by RT-qPCR 14d after BDL operation The expressions of lncRNA NONRATT002335 and NONRATT018085 were significantly up-regulated underlying BDL group compared with control group; the expression of lncRNA NONRATT025415, NONRATT025388 and NONRATT025409 was significantly down-regulated underlying BDL group compared with control group whereas the expression of lncRNA NONRATT02538 and NONRATT006517 had not statistically different between control group and BDL group.

**Figure 7:**
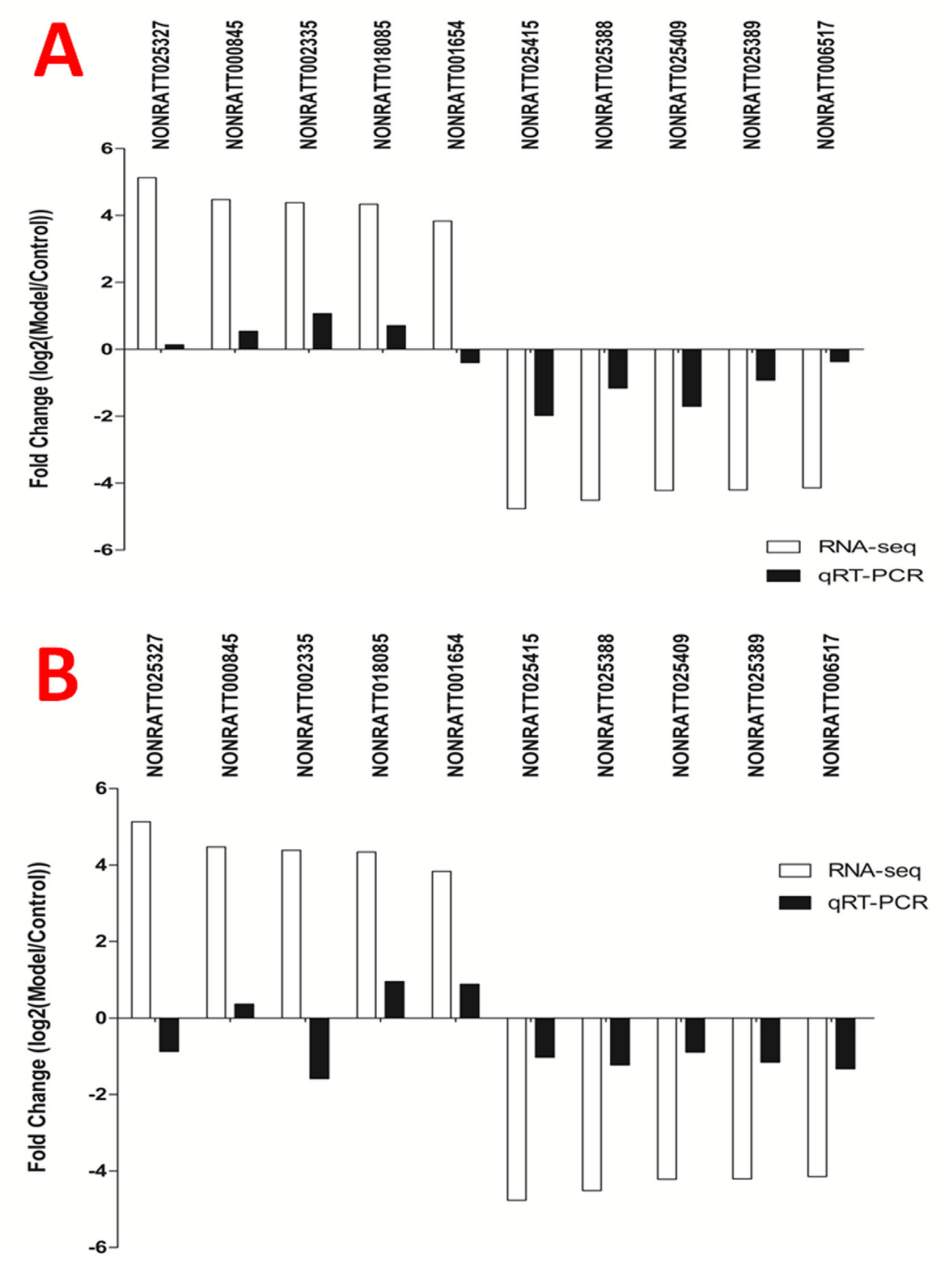
The expression of 10 lncRNAs in T6-T12 spinal cord at different time points after BDL operation **(A)** 14d after BDL operation, four up-regulated lncRNAs and five down-regulated lncRNAs were validated by RT-qPCR. **(B)** 28 d after BDL operation, three up-regulated lncRNAs and five down-regulated lncRNAs were validated by RT-qPCR.

Obviously, gene expressions after BDL operation injection are varied in different time points. We collected spinal tissue sample from T6-T12 spinal cord 14d vs 28d after BDL operation for RT-qPCR validation. 14d after BDL operation, the expressions of lncRNA NONRATT002335 and NONRATT018085 were significantly up-regulated underlying BDL group compared with control group whereas the expression of lncRNA NONRATT025327, NONRATT000845 and NONRATT001654 had not statistically different between control group and BDL group; The expression of lncRNA NONRATT025415, NONRATT025388 and NONRATT025409 was significantly down-regulated underlying BDL group compared with control group whereas the expression of lncRNA NONRATT02538 and NONRATT006517 had not statistically different between control group and BDL group (Figures 6 and 7).

## DISCUSSION

In the present study, we confirmed that bile duct ligation resulted in obstructive cholestasis and jaundice and this model was consistent with previous reports describing marked elevation of the serum total bilirubin in rats responding to bile duct ligation [5, 25]. In addition, our result also indicated that obstructive jaundice induced the decreased sensitivity in response to the mechanical nociceptive stimulation 14d and 28d after BDL operation, which was in line with a previous experimental study showing that obstructive cholestasis by bile duct resection in rodents displayed decreased nociception [26–28].

We identified the spinal genes and lncRNAs that were differentially expressed between model group and control group by high throughput RNA-seq, providing an important view of spinal genetic heterogeneity for jaundice-induced decreased nociception in rats. The present study identified 2033 differentially expressed lncRNAs in the spinal cord, 488 of which were down-regulated >2-fold whereas 1545 of which were up-regulated >2-fold. Similarly, a total of 2800 mRNAs were differentially expressed between control group and BDL group, 1252 of which were down-regulated whereas 1548 of which were up-regulated (FDR<0.05, |log2FC|>1). Among top 5 up-regulated lncRNAs and top 5 down-regulated lncRNAs, 2 up-regulated (NONRATT002335 and NONRATT018085) and 3 down-regulated lncRNAs (NONRATT025415, NONRATT025388 and NONRATT025409) were identified. These results suggested that 50% differentially expressed lncRNAs were verified by RT-qPCR. Furthermore, we found that Serpina3n was an important up-regulated gene 28d after BDL operation, which were in agreement with a previous study that the serine protease inhibitor Serpina3n was up-regulated in the dorsal root ganglia (DRG) after nerve injury, and attenuated neuropathic pain by inhibiting T cell-derived leukocyte elastase [29].

Previous studies have suggested that the different duration of cholestasis accompanies by some behavioral change in rodents [26, 30]. Belghiti et al observed that chronic BDL rats displayed enhanced scratching behavior and thermal hyperalgesia already 48 h after BDL surgery, and this result was perfectly demonstrated by the up-regulation and sensitization of the heat-sensitive TRPV1 channel [30]. Tian et al also demonstrated 5-HT-induced enhanced scratching and antinociception to mechanical and heat stimuli in BDL rats [31], suggesting that targeting 5-HT receptors may be an effective treatment for cholestatic itch. In this study, we found differential expression of some lncRNAs (Figure 7) in the spinal cord at different time points (14d/28d) after BDL operation, suggesting that the different lncRNAs in spinal cord segment may be involved in the neuronal response to obstructive jaundice. Although the functions of many lncRNAs in spinal cord are not fully known, our findings provide novel potential insights involving in the molecular mechanism of jaundice-induced altered peripheral nociception.

In conclusion, obstructive jaundice accompanied by altered peripheral nociception is always a crucial factor limiting the therapeutic efficacy of many drugs and quality of life for patients. Here we constructed the expression profiles of lncRNAs and potentially related mRNAs in rats with obstructive jaundice, and found some distinct lncRNA/mRNA expression profiles in spinal cord, suggesting that these unique noncoding transcripts may contribute to the acquisition of cholestasis-induced altered peripheral nociception. Although additional studies are needed to verify these lncRNAs/mRNAs mentioned above, our study provides important insights into novel indicators of treatment for patients with cholestasis.

## MATERIALS AND METHODS

### Animal care

Male Sprague-Dawley rats were provided by the Center of Experimental Animal of Tongji Medical College (license number: 43004700019962). All experimental protocols, animal testing and surgeries were performed in adherence to the National Institute of Health Guide for the Care and Use of Laboratory Animals (NIH Publications No. 80-23) revised 1996. The experimental protocols were approved by the committee of experimental animals of Tongji Hospital, Tongji Medical College (IRBID:TJ-A0804). The animals were maintained in a climate controlled room on a 12-hlight/dark cycle (light on at 07:00 h). Rats were housed (2/cage), but they were individually caged during each experiment. Food and water were available *ad libitum*.

### Obstructive jaundice model by bile duct ligation

Rats were placed in a temperature-controlled chamber before experimental operation. All surgical procedures were performed under sterile conditions. Common bile duct ligation (BDL) established the prolonged obstructive jaundice as described in previous studies [32–34]. After an intraperitoneal injection of ketamine hydrochloride (50 mg/kg) plus xylazine (5 mg/kg), rats which underwent BDL were anesthetized. In those with BDL, the common bile duct was located and ligated using 4-0 silk at two points anterior to the pancreas and posterior to the hilum of the liver. The first ligation was made just above the duodenum and the second almost 2 mm above the first ligation. The bile duct was then transected at the midpoint between the two ligatures. Animals used in this study were sacrificed before signs of severe illness became apparent.

### Experimental groups

**Experiment A** Rats were randomly assigned to two groups: (1) Control group (sham-ligation surgery, n = 9); (2) BDL group (common bile duct ligation, n = 9). The mechanical nociceptive thresholds were evaluated at different time points including day (d) 0 (baseline), 3, 7, 14, 21, and 28. 28d after operation, rats were sacrificed, and blood samples, spinal cord tissues were collected for further analysis. Serum total and direct bilirubin levels were measured. Thoracic segments of spinal cord (T6-T12) were prepared to analyze differential gene and lncRNA expression patterns by high-throughput RNA sequencing, and thoracic (T6-T12) segment of spinal cord for Real-Time quantitative PCR (RT-qPCR).

**Experiment B** Rats were randomly assigned to two groups: (1) Control group (n = 12); (2) BDL group (n = 12). 14 days after surgery the T6-T12 spinal cord were collected using a dissection microscope, rinsed with isotonic saline, dissected and fleshly frozen in liquid nitrogen for RT-qPCR.

### Assessment of mechanical sensitivity

As in previous reports [35–38], mechanical paw withdraw threshold was examined using the blind method with a minimum of six animals/group. Calibrated von Frey filament apparatus (Stoelting, Wood Dale, IL, USA) was used to measure the paw withdrawal response for a mechanical stimulus. A range of filament comprises 1g, 1.4g, 2g, 4g, 6g, 8g, 10g, and 15g bending force. Rats were allowed to acclimate within a clear plastic chamber over an elevated mesh floor for 30 minutes before assessment at room temperature. Each filament was applied to the midplantar surface of the left hind paw until a withdrawal response occurred. To avoid tissue injury in refractory animals, stimulation was automatically terminated after 15 seconds. The interval between adjacent tests was >5 minutes. The results of 3 consecutive measurements were averaged for the mechanical paw withdraw threshold in BDL and control rats.

### Analysis of jaundice index

28d following BDL operation, serum total bilirubin levels were determined in the Automated Blood Chemical Analyzer Vitro 350 (Orthoclinical Diagnostic Inc., Rochester, NY).

### RNA extraction, library preparation and high-throughput RNA sequencing

RNA was isolated from the thoracic (T6-T12) spinal segment tissues in two groups using TRI reagent (Invitrogen, Carlsbad, CA, USA) according to the manufacturer's instructions. Tissue samples were determined using a NanoDrop 2000 (Thermo Fisher Scientific Inc, USA) for integrity, quality and purity.

Library preparation and high-throughput RNA sequencing were performed by the OE Biotechnology Corporation (Shanghai, China) [39–41]. Total RNA of all samples were sequenced by EMBL GeneCore (EMBL Genomics Core Facility, Heidelberg, Germany). The synthesis of polyadenylated transcriptome libraries for every sample was accompanied by deep sequencing in 3 lanes each generating >50 million reads pair end. RNA-seq was performed in the two groups, each with three biological replicates.

### Real-time quantitative-PCR

Thoracic (T6-T12) segment of spinal cord were dissected and isolated, and total RNA was extracted using TRIzol reagent (Beijing Tiangen Biotech CO.). 6 μg total RNA was used as a template for cDNA synthesis and amplification using the First-Stand Synthesis System (BioPhotometer, Eppendorf, Hamburg, Germany) according to the manufacturer's instructions [35, 42–45]. The primers were designed with the Primer Express 3.0 software (Applied Biosystems), and the specific forward (F) and reverse (R) primer sequences were in Table 4. Experiments were evaluated in triplicate and repeated at least three times. The threshold cycle (CT) was used to estimate the amount of target mRNA. The comparative CT method with the formula for relative fold-change = 2^−ΔΔCT^ was used to quantify the amplified transcripts.

**Table 4:** Primer sequences for RT-qPCR

Gene	Forward (5′ to 3′)	Reversed (5′ to 3′)
NONRATT025327	ATGCACCGCTAGAAGTCACAGA	GCTCAGACCCAGTTGCTCCC
NONRATT000845	AGTAAACCCCTAACAAATCCCC	GGTCACCTAAGACCATTGGAAA
NONRATT002335	AGCCATGCGTCTTCCTAACC	AGCACCTGAACAAGCCACCT
NONRATT018085	ACCAGATGGAACGATTAAACCC	CCACTGCCACTGAACCTTGA
NONRATT001654	GGGTGGCTGGATTTCATTTT	GGGGAACAGAGGGAATACAACA
NONRATT025415	CTGGTCTGGGTTTTCCTTCTTG	TGAGGTGGCACAGGTGAGTTT
NONRATT025388	TCCACTGATTTCCAGGCTCTT	GCCCTTCACTTGACACCTACAC
NONRATT025409	AGGGCTTGAAGTGGATGGG	TTGCAGTGCTGGCAGAGG
NONRATT025389	ATTGACTCTGGCCTGGGAGC	TTATGGTGCAAGTGAGGTTGG
NONRATT006517	ATCCAAAATGATTTCCTACCCA	GACCAGCTATAAGCCAGTGTCC
GAPDH	CGCTAACATCAAATGGGGTG	TTGCTGACAATCTTGAGGGAG

RT-qPCR: reverse transcriptase quantitative polymerase chain reaction

### Data analysis

Results are expressed as the mean ± SEM. Statistical comparisons were performed with Mann-Whitney test. Statistical comparisons were performed using unpaired or paired Student's t tests or two-way repeated measures ANOVA. P < 0.05, p < 0.01, and p < 0.001 represent statistically significant differences.

## Abbreviations

BDL: bile duct ligation
GO: gene ontology
KEGG: Kyoto Encyclopedia of Genes and Genomes
SD: Sprague-Dawley
RT-qPCR: quantitative real-time PCR
DEGs: differentially expressed genes
lncRNAs: long noncoding RNAs
